# Phenotypic characterization of the *Hordeum bulbosum* derived leaf rust resistance genes *Rph22* and *Rph26* in barley

**DOI:** 10.1111/jam.15710

**Published:** 2022-07-26

**Authors:** Xiaohui Yu, Seona Casonato, Eirian Jones, Ruth C. Butler, Paul A. Johnston, Soonie Chng

**Affiliations:** ^1^ Faculty of Agriculture and Life Sciences, Department of Pest‐Management and Conservation Lincoln University Lincoln, Canterbury New Zealand; ^2^ The New Zealand Institute for Plant and Food Research Limited Lincoln, Canterbury New Zealand; ^3^ StatsWork 2022 Limited Lincoln, Canterbury New Zealand

## Abstract

**Aims:**

Two introgression lines (ILs), 182Q20 and 200A12, which had chromosomal segments introgressed from *Hordeum bulbosum* in *H. vulgare* backgrounds, were identified to show seedling resistance against *Puccinia hordei*, possibly attributed to two resistance genes, *Rph22* and *Rph26*, respectively. This study characterized the phenotypic responses of the two genes against *P. hordei* over different plant development stages.

**Methods and Results:**

Using visual and fungal biomass assessments, responses of ILs 182Q20, 200A12 and four other barley cultivars against *P. hordei* were determined at seedling, tillering, stem elongation and booting stages. Plants carrying either *Rph22* or *Rph26* were found to confer gradually increasing resistance over the course of different development stages, with partial resistant phenotypes (i.e. prolonged rust latency periods, reduced uredinia numbers but with susceptible infection types) observed at seedling stage and adult plant resistance (APR) at booting stage. A definitive switch between the two types of resistance occurred at tillering stage.

**Conclusions:**

*Rph22* and *Rph26* derived from *H. bulbosum* were well characterized and had typical APR phenotypes against *P. hordei*.

**Significance and Impact of the Study:**

This study provides important insights on the effectiveness and expression of *Rph22* and *Rph26* against *P. hordei* during plant development and underpins future barley breeding programmes using non‐host as a genetic resource for leaf rust management.

## INTRODUCTION

Leaf rust caused by *Puccinia hordei* is one of the most important diseases of cultivated barley (*Hordeum vulgare* L.) and is widely distributed in major growing areas worldwide (Clifford, 1985). Depending on different regions, years and disease pressures, yield losses of around 10%–45% due to the disease have been reported (Griffey et al., 1994; Murray & Brennan, 2010; Teng & Close, 1978; Wright & Gaunt, 1992). To reduce yield losses caused by leaf rust, breeding elite barley cultivars with effective disease resistance is considered the most cost‐effective and ecologically sustainable approach (Park et al., 2015). In general, plant host resistance can be classified as either qualitative or quantitative resistance. Qualitative resistance induces hypersensitive response and localized cell death to suppress the advancement of the pathogen (Kushalappa et al., 2016). Qualitative resistance is usually mediated by single receptor‐encoding *R* genes, and is only effective on specialized pathogens with compatible effectors encoded by specific avirulence (*Avr*) genes (Jones & Dangl, 2006; Sarris et al., 2015). Due to the monogenic nature of qualitative resistance and the rapid development of new virulent pathotypes via selection pressure, sexual recombination or step‐wise mutation, a large number of these resistance genes have been rendered ineffective against *P. hordei* (Park, 2003; Park et al., 2015).

Qualitative resistance is often expressed at all growth stages (GSs) of plant development, and the hypersensitive response is easily identified at seedling stage in a greenhouse; thus, the terms of all stage resistance and seedling resistance have been widely used to refer to the qualitative resistance to *P. hordei*. On the other hand, quantitative resistance tends to be controlled by a number of quantitative trait loci and ends up with a continuous distribution of phenotypes with reduced disease severity rather than complete resistance in a population (St.Clair, 2010). Quantitative resistance is considered to be more durable, because multiple genetic changes in single pathogens are required to occur concurrently to break it down (Lo Iacono et al., 2012). Meanwhile, the polygenic nature makes quantitative resistance difficult to characterize and utilize. Because of the phenotype of reduced susceptibility, quantitative resistance is also called partial or slow‐rusting resistance. Another form of host resistance referred to as adult plant resistance (APR), which is only effective at post‐seedling GSs, also confers the same disease response as quantitative resistance (i.e. incomplete resistance). In addition, some partially resistant cultivars that were characterized had shown effective APR in field tests (Golegaonkar et al., 2009). Thus, the terms, quantitative resistance, partial resistance, slow‐rust resistance and APR to *P. hordei*, have been used interchangeably (Derevnina et al., 2013).

A plant species is classified as a non‐host to a given pathogen if all accessions of the plant species have uniform levels of resistance against all isolates of the pathogen, which is virtually impossible to test in practice (Bettgenhaeuser et al., 2014). Thus, identification of a non‐host is usually based on common experience or tests with a limited number of pathogen isolates (Panstruga & Moscou, 2020). Based on its definition, non‐host resistance is considered to be durable. However, hybridization barriers usually occur between the non‐host and host plant species that the given pathogen is able to colonize, which dramatically hampered the study of genetic components and their underlying mechanisms contributing to non‐host resistance. Considerable evidence indicates that *Hordeum bulbosum* is a non‐host to *P. hordei* (Anikster, 1989; Pickering et al., 1998; Pickering et al., 2000). Interspecific crosses between *H. bulbosum* and *H. vulgare* can produce partially fertile hybrids that give rise to introgression lines (ILs), which possess *H. bulbosum* introgressions in *H. vulgare* backgrounds (Szigat & Pohler, 1982). These ILs are valuable resources for uncovering components of non‐host resistance against *P. hordei*. In addition, *H. bulbosum*, as the sole member of the secondary gene pool of cultivated barley, is another potential genetic resource of novel disease resistance genes for barley improvement (Fetch Jr et al., 2009; Pickering et al., 1995; Xu & Kasha, 1992).

To date, 27 *Rph* (resistance to *P. hordei*) genes have been genetically characterized and formally designated. Of these, 23 were identified from the primary gene pool of barley, including *H. vulgare* subsp. *vulgare* (cultivated barley) and *H. vulgare* subsp. *spontaneum* (wild barley). Only four *Rph* genes (*Rph17*, *Rph18*, *Rph22* and *Rph26*) have been derived from *H. bulbosum*. Previous study by Pickering et al. (2004) on *H. bulbosum* ILs had identified ILs 182Q20 and 200A12 to show partially resistant phenotypes, such as prolonged rust latency periods (LPs; i.e. the time to 50% final uredinium formation) and reduced infection frequencies (uredinia number cm^−2^), against *P. hordei* at seedling stage. In that study, 182Q20 and 200A12 were designated G‐2HL‐b and E‐1HL, respectively. The resistance genes *Rph22* (carried by the IL 182Q20) and *Rph26* (carried by the IL 200A12) were genetically mapped to the respective barley chromosomes 2HL and 1HL within the introgressed *H. bulbosum* segments (Johnston et al., 2013; Yu et al., 2018). Although partial resistance at seedling stage has been reported (Pickering et al., 2004), the pathological characteristics of these two resistance genes against *P. hordei* over the course of different plant development stages are still unknown. In this study, the phenotypic responses of two ILs carrying either *P. hordei* resistance gene, *Rph22* or *Rph26*, over the course of different plant development stages were characterized and compared to their backgrounds using conventional visual assessment and novel internal fungal growth quantification methods (Bettgenhaeuser et al., 2014).

## MATERIALS AND METHODS

### Selected barley lines and inoculum

The two ILs, 200A12 and 182Q20, and four barley cultivars, ‘Emir’, ‘Golden Promise’, ‘Gus’ and ‘Vada’, were initially evaluated for their responses to *P. hordei* at seedling and subsequently booting (adult plant) stages using the same plants in a greenhouse. The resulting phenotypic responses guided a follow‐up experiment to examine the responses of the two ILs, ‘Emir’ and ‘Golden Promise’ at tillering and stem elongation stages against the pathogen. Before inoculation, the actual plant growth stage (GS) was assigned a Zadoks decimal code (Zadoks et al., 1974). Cultivars ‘Emir’ and ‘Golden Promise’ are the barley parents of ILs 200A12 and 182Q20, respectively, which means that except for the introgressed *H. bulbosum* segments, both ILs 200A12 and 182Q20 are genetically identical to their parents. A study by Wendler et al. (2015) has confirmed that both 182Q20 and 200A12 (samples 84 and 86, respectively) have only one *H. bulbosum* introgression. Hence, any difference in phenotypic response against *P. hordei* between the ILs and their parents could be attributed directly to the resistance genes *Rph22* and *Rph26*. The cultivar ‘Gus’ is susceptible to *P. hordei* due to the lack of any resistance, while ‘Vada’ is known to have partially resistant phenotypes against *P. hordei* (Parlevliet, 1975). Pre‐germinated seedlings were transplanted into either trays or pots (depending on experiments) containing a potting mix (shredded bark and washed crushed sand in a ratio of 3:2) supplemented with 6.4 kg m^−3^ dolomite lime, 3.32 kg m^−3^ ‘Osmocote 14‐16.1‐11.6’, 104 kg m^−3^ superphosphate, 120 g m^−3^ calcium nitrate, 56 g m^−3^ ‘FetrilonCombil’ (BASF) and 1 kg m^−3^ ‘Micromax’ (micronutrients, SealesWinslow, New Zealand). The seedlings were maintained at 20°C in a greenhouse until inoculation. A single *P. hordei* isolate (BLR‐14/11) (Plant & Food Research, Lincoln) was used for all the experiments. This isolate was virulent to barley leaf rust resistance genes *Rph1*, *Rph2*, *Rph3*, *Rph4*, *Rph6*, *Rph9*, *Rph10*, *Rph12* and *Rph19* (unpublished data) using International and Australian series of differential genotypes after the methods of Park (2003). “The isolate BLR‐14/11 was collected in 2011 from an infected barley crop, was never exposed to any breeding materials consisting Rph 22 and 26 in the field, thus it likely would not be virulent to the two resistance genes.”

### Disease development in a greenhouse

#### Seedling stage

The seedling stage experiment, which consisted of the six barley lines, was carried out in rectangular trays (35 × 20 × 5 cm) using a resolvable block design generated with CycSoftware 2009 (CycDesigN 4.0, CycSoftware Ltd, New Zealand). Each tray (block) had three barley lines (one seedling per line) sown along one lengthwise side, with two trays forming a complete replicate. Each line was replicated five times. Additional seedlings of each barley line were transplanted into pots and used as uninoculated controls for fungal biomass assessment. To ensure uniform spore coverage during inoculation, the adaxial surface of leaf‐2 from each plant at GS 12 (two‐leaf stage) was secured flatly onto a plastic board (40 × 30 cm) at the ends with masking tapes. Inoculations were carried out by atomizing a urediniospore suspension (1.5 mg urediniospores 1.5 ml^−1^ mineral oil [Pegasol, Mobil Oil] per replicate) onto the secured leaves using an airbrush. Urediniospore deposition and percentage germination were estimated by placing water agar slides (2%) on the plastic boards prior to inoculation, with three water agar slides per replicate. Inoculated seedlings and water agar slides were incubated at 22°C in the dark at 100% relative humidity for 24 h. The seedlings were then maintained at 22 ± 2°C in a greenhouse. Spore deposition and percentage germination were determined after counting the number of germinated and non‐germinated urediniospores within a randomly selected 1 cm^2^ areas on the water agar slides under a compound microscope at 100× magnification. The urediniospores with germ tubes equal to or greater than the width of spores were considered as germinated.

#### Tillering and stem elongation stages

Four barley lines, 200A12, 182Q20, ‘Emir’ and ‘Golden Promise’, were examined at tillering (GS 21) and stem elongation (GS 32) stages. Pots were prepared with a single seedling of a given line, with three replicates for each of the line by GS combinations, giving a total of 24 plants or pots. Pots were arranged according to a Latinized resolvable row–column design. Extra seedlings of each barley line were also grown as uninoculated controls and for fungal biomass assessment. Inoculations at tillering and stem elongation stages were carried out at GS 21 (main shoot and one tiller detectable) and 32 (second node detectable), respectively. To minimize the influence of leaf maturity on plant responses to *P. hordei*, only the uppermost leaves that were fully expanded on the main shoots were inoculated for disease assessment (2 mg urediniospores 2 ml^−1^ mineral oil for each replicate). Methods on inoculation, estimation of spore deposition and germination, and plant incubation conditions were similar to those described for the seedling experiment.

#### Booting stage

The same plants from the seedling experiment were used for the adult plant study (booting stage). In the current study, six plants from each line were transplanted into 4‐L pots (15 cm in diameter) with one plant per pot. Therefore, each line was replicated six times. The plants were arranged according to a randomized complete block design. They were inoculated at GS 41 when flag leaf sheaths were extending. Each replicate was inoculated separately (2 mg of urediniospores 2 ml^−1^ mineral oil) before maintaining at similar conditions as the seedling experiment.

### Disease assessments

#### Seedling, tillering and stem elongation stages

Similar methods were used to assess leaf rust at seedling, tillering and stem elongation stages, in which only the selected leaves were secured flatly for inoculations. The number of uredinia within a marked 5 cm long leaf area was counted daily until 13–14 days post inoculation (DPI). Rust LP was determined as the time from inoculation to the time when 50% uredinia appeared. Infection type (IT) was scored at 10 DPI using a 0–4 scale described by Park (2003). ITs of 3+ or higher were considered compatible (i.e. virulent pathogen/susceptible host). The visual assessments included counts of uredinia, rust LP (days) and IT.

After visual assessment, fugal biomass was quantified using a modified method (Ayliffe et al., 2014) as described in Yu et al. (2018). In this method, 5 cm long leaf material from all the replicates of each line and the uninoculated controls were pooled and processed separately before three sub‐samples were taken from each for biomass quantification. As leaf rust was the only disease seen on the infected plants, it was assumed that only *P. hordei* was quantified. The net fungal biomass of each test barley line was obtained by subtracting the average fluorescence reading of non‐infected leaf sample from that of infected leaf sample.

#### Booting stage

For the adult plant experiment, only the first and second leaves below the flag leaf on the main tiller, coded as Flag‐1 and Flag‐2 respectively, were assessed for disease response at 8 and 14 DPI. A modified Cobb scale (Peterson et al., 1948; cited in Golegaonkar et al., 2009) was used to score disease severity (percentage of leaf area covered with uredinia) and host responses (R, no uredinia present; TR, trace or minute uredinia on leaves without sporulation; MR, small uredinia with slight sporulation; MR–MS, small‐to‐medium‐sized uredinia with moderate sporulation; MS–S, medium‐sized uredinia with moderate to heavy sporulation; S, large uredinia with abundant sporulation) were scored after the methods outlined by McIntosh et al. (1995).

### Statistical analyses

#### Uredinia counts and fungal biomass from seedling, tillering and stem elongation experiments

The final uredinia counts at 13 (or 14) DPI were analysed with a hierarchical generalized linear model (GLM) approach (Lee et al., 2006), with a Poisson distribution for the fixed effects (barley lines) and a gamma distribution for random effects (replicates), with logarithmic links for both. The dispersion was estimated. The importance of the random effects was assessed with a *χ* ^2^ test of the change in deviance on dropping the term, as implemented in Genstat's HGRTEST procedure (VSN International Ltd, 2015). An overall test for differences between the barley lines was carried out similarly, using Genstat's HGFTEST procedure. In the results, means and associated 95% confidence limits are presented. These were obtained on the link (logarithmic) scale, and back‐transformed for presentation.

Analysis of cumulative number of uredinia over time was carried out in two parts: first, the total uredinia per barley line at each assessment time was analysed; second, the number of uredinia per barley line per replicate at each assessment was analysed. When combined with the results from the first analysis, this second analysis allowed an assessment of whether there was substantial variation between replicates within barley lines. The distribution of times to uredinia formation was modelled using the logistic distribution of the log days after inoculation, as implemented in Genstat's CUMDISTRIBUTION procedure (Butler et al. in VSN International Ltd, 2015). It was assumed that no further uredinia would be formed after the final assessment (13 DPI). The cumulative distribution function (c.d.f) applied was *F*(*z*):

$$F\left(z\right)=\frac{1}{1+e^{\left(-b\times \left(z-m\right)\right)}}\;$$

where *z* = log(DPI ‐ Lag), and *b*, *m* and Lag are estimated parameters.

The primary results of these analyses were estimates of the lag before uredinia began to appear (Lag), the mean time to uredinia formation (Mean), the time to 50% uredinia formation (*T*50) and the standard deviation of the distribution of times to uredinia formation (*SD*). Mean, T50 and *SD* were calculated from Lag, *b* and *m*, as follows:

$$\begin{array}{l} \text{Mean}=\mathrm{Lag}+\frac{\pi }{b}\times \frac{e^{m}}{\sin\left(\pi /b\right)}\\ T50=\mathrm{Lag}+e^{m}\\ \mathrm{SD}=e^{m}\times \sqrt{\frac{\pi }{b\times \sin\left(\pi /b\right)}\times \left(\frac{1}{\cos\left(\pi /b\right)}-\frac{\pi }{b\times \sin\left(\pi /b\right)}\right)}. \end{array}$$

Some ‘analysis of parallelism’ (Ross, 1984) was carried out to assess whether any of the parameters *b*, *m* or Lag varied substantially between barley lines. The test statistics used were Seedlings: $X_{5}^{2}$=17.7, *p* = 0.003; to compare between the model with T50 the same for all barley lines, and the model with T50 varying between barley lines; GS 21 and 32: $X_{7}^{2}$=24.8, *p* = 0.001 to compare between the model with T50 the same for all lines at both stages, and T50 varying between lines and GSs.

Fungal biomass data for all the three GSs were analysed together. The data were analysed using a mixed‐model analysis, carried out with REML (Payne et al., 2017). Fixed effects included barley lines, GS, infected versus uninoculated and interactions among these. The variability between sub‐samples from uninoculated plant samples was much smaller than that for samples from the inoculated plants, so separate variances were estimated within the analysis for each. Since the leaf material was pooled across replicate leaves and then sub‐sampled, it was not possible to obtain an estimate of the between leaf variation. However, pseudo ‘*F*' test statistics (but not valid *p*‐values) based on the within‐pooled sample variation could be used to assess the relative importance of the factors. To assess the resistance response of ILs over plant development, an indirect parameter – relative resistance expression, based on percentage decreases in uredinia number and fungal biomass (%), and increase in rust LP (%) of ILs to their barley parents, at each GS was used. Higher reduction in uredinia number or fungal biomass, or higher increase in rust LP indicated a stronger relative resistance conferred by the IL to its barley background at a given GS.

#### Host response data from adult plant stage experiment

The six host response types R, MR, MR–MS, MS, MS–S and S were converted to values of 0.15, 0.30, 0.45, 0.60, 0.75 and 1.0, respectively. A coefficient of infection (CI) for each inoculated leaf was calculated by multiplying its host response value by final disease severity (Golegaonkar et al., 2009). Both severity and CI were analysed with a binomial GLM, with a logit link (McCullagh & Nelder, 1989), a binomial total of 100, and the dispersion estimated. The various effects – barley lines, leaves, DPI, and the interactions between these – were assessed with *F*‐tests, within the analyses of deviance done as part of the analyses. Percentages and mean CI for each barley line, leaf and DPI combination and associated 95% confidence limits were obtained on the link (logit) scale, and converted to percentages. The mean CI for each leaf and assessment time is the average coefficient of infection (ACI). Barley lines with ACI scores of 0–7, 8–16 and 17–24 at 14 DPI were considered to have high, moderate and low APR to *P. hordei*, respectively. While for those having final ACI scores of 25 and above, they were considered as lacking useful APR and were classified into a susceptible group (Kumar et al., 2019). This classification was applicable to the current study since the focus was to compare the two ILs 200A12 and 182Q20 against their parental backgrounds (Kumar et al., 2019). In the case of screening a large number of breeding lines, ACI values of 21–40 might be considered as carrying minor resistance genes against leaf rust (Golegaonkar et al., 2009). All analyses were carried out with Genstat (Payne et al., 2017).

## RESULTS

### Uredinia development and biomass accumulation during seedling, tillering and stem elongation stages

**FIGURE 1:**
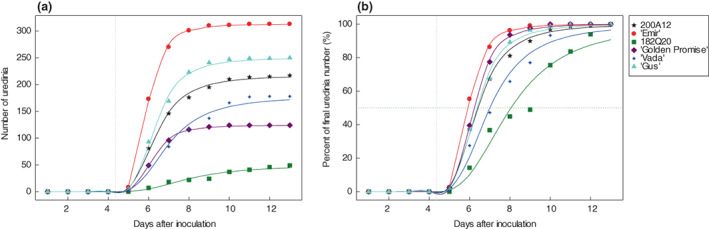
Cumulative number of uredinia (a) and percentage of final uredinia number (b) developed days after inoculation for the six barley lines at seedling stage. The vertical dotted line shows the end of the lag phase. The horizontal dotted line indicates the appearance of 50% of the final number of uredinia.

Final uredinia number per replicate varied significantly between the test barley lines at each GS (*p* < 0.001). The IL 182Q20 had the lowest uredinia numbers at GS 12 and GS 21, and the second lowest at GS 32 among the test barley lines (Table 1). For IL 200A12, although a high number of uredinia developed (but still 30.7% less than ‘Emir’) at GS 12, the number was the second lowest and the lowest at GS 21 and GS 32, respectively (Table 1). Cultivar ‘Golden Promise’ had slightly more uredinia than ‘Emir’ at GS 32, but ‘Emir’ was considered the most susceptible line to *P. hordei* due to its continued high uredinia numbers over the remaining GSs. Over time, uredinia developed more slowly in IL 182Q20 and resulted in the longest LPs (T50) at every GS compared with other barley lines (Table 1; Figure 1). The partially resistant cultivar ‘Vada’ had the second longest LP for *P. hordei* at GS 12 followed by IL 200A12. At GS 21 and 32, where cultivar ‘Vada’ was not included, IL 200A12 had the second longest LP after IL182Q20 (Table 1). In terms of rust LP, cultivar ‘Emir’ was still considered the most susceptible barley line as ‘Emir’ reached its 50% final uredinia formation in the shortest time frame at every test GS compared with other barley lines (Table 1; Figure 1). All the test barley lines gave compatible responses to *P. hordei* at seedling stage (Table 1) with no distinctive differences between the ILs and their barley parents. At GS 21 and 32, both ILs showed limited development of uredinia, which was characterized by reduced uredinia number and size.

**TABLE 1:** Phenotypic responses^a^ of the six test barley lines to *Puccinia hordei* assessed by mean final number of uredinia (95% confidence limits), rust latency period (LP) (standard errors), and infection type (IT) at growth stages (GSs) 12 (seedling), 21 (tillering) and 32 (stem elongation)

Barley line	GS 12 (seedling)			GS 21 (tillering)			GS 32 (stem elongation)		
	Mean final uredinia number	LP	IT	Final uredinia number per replicate	LP	IT	Mean final uredinia number	LP	IT
‘Emir’	78.2 (36.9, 165.8)	5.93 (0.06)	3 + C	54.3 (30.0, 98.3)	6.21 (0.07)	3 + C	98.7 (63.6, 153.1)	6.30 (0.05)	3 + C
200A12	54.2 (25.5, 115.4)	6.40 (0.12)	3 + C	18.0 (6.4, 50.4)	6.35 (0.11)	2	6.3 (1.1, 35.9)	7.64 (0.41)	2−
‘Golden Promise’	35.4 (16.5, 76.2)	6.21 (0.12)	3+	45.0 (23.5, 86.3)	6.25 (0.07)	3+	101.3 (65.7, 156.4)	6.31 (0.04)	3+
182Q20	13.9 (6.2, 31.0)	8.06 (0.50)	3+	10.3 (2.7, 40.1)	8.65 (0.53)	2−	12.3 (3.6, 42.8)	9.11 (0.19)	2−
‘Gus’	62.5 (29.4, 132.7)	6.35 (0.10)	4	—	—	—	—	—	—
‘Vada’	44.5 (20.8, 95.0)	7.01 (0.18)	3+	—	—	—	—	—	—

*Note*: IT was determined after the methods of Park (2003).

a ‘—’ = not included in the experiments.

**FIGURE 2:**
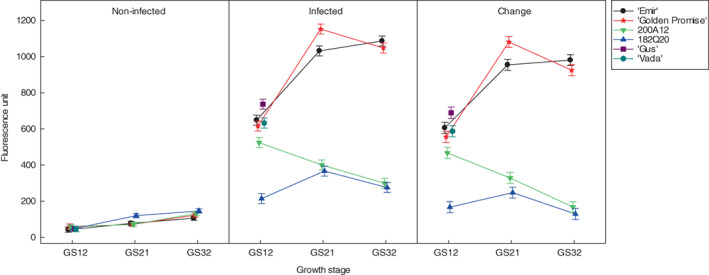
Mean fluorescence units of the six non‐infected and infected test barley lines by *Puccinia hordei*, and their net change at growth stages 12 (seedling), 21 (tillering) and 32 (stem elongation). Error bars are 95% confidence limits for the means estimated from the pooled between‐subsample variation for samples combined across replicates.

As expected, fungal biomass was substantially larger for infected leaves than for non‐infected leaves (Figure 2; ‘*F*’ = 19,183.4). However, the fluorescence reading from infected leaves varied between the lines (‘*F*’ = 1032 for the Treatment by Line interaction): At a given GS, the fluorescence reading of non‐infected leaves was relatively similar across the lines (‘*F*’ = 1, 17, 9 for seedling, GS21 and GS32, respectively), but varied more for infected plants (‘*F*’ = 178, 900, 1075). In particular, fluorescence for infected plants was considerable lower for lines 200A12 and 182Q20 at stages GS21 and GS32 than it was for the other lines (Figure 2).

The net internal fungal growth of test barley lines was highly correlated with the mean final uredinia number at GS 21 (*r* = 0.95) and GS 32 (*r* = 0.99). However, a couple of lines that had high uredinia number and fungal biomass influenced the correlations at each GS. At GS 12, a moderate correlation (*r* = 0.78) between the two parameters was obtained with a notable conflicting result observed in IL 200A12 (Figure 2).

#### Relative resistance expression over GSs

At the three test GSs, both ILs 182Q20 and 200A12 had decreased fungal biomass and uredinia number, and increased rust LP compared with their barley parents (Table 1; Figure 2). For these three parameters, an increasing resistance to *P. hordei* was also observed over different plant development stages for both ILs 182Q20 and 200A12 (Table 2). An exception was for IL 200A12 at GS 21, which had a very similar rust LP to ‘Emir’ under similar experimental conditions. Despite having a compatible ITs at seedling stage, IL 182Q20 showed a strong relative resistance expression based on its 60.7% and 70.0% reductions in uredinia number and internal fungal biomass, respectively, and 29.8% increase in rust LP compared with ‘Golden Promise’. The expression of resistance increased over the GSs and culminated with over 85.0% reductions in uredinia number and internal fungal biomass, and 44.4% increase in fungal LP at GS 32. While for IL 200A12, only 22.9% and 30.7% reductions in fungal biomass and uredinia number, and 7.9% increase in rust LP at seedling stage, relative to ‘Emir’. The resistance, however, increased drastically at GS 32 with greater than 80% reduction in fungal biomass and uredinia number, and increased rust LP by 21.3%.

**TABLE 2:** Relative resistance expressions of introgression lines 200A12 and 182Q20 at each growth stage (GS) to *Puccinia hordei* based on percentage reduction (−) in uredinia number and fungal biomass fluorescence unit, and increase (+) in rust latency period (LP) compared with their barley parents. A higher percentage value indicates a higher expression of resistance

IL	Resistance measurement	Relative resistance expression (%)		
		GS 12 (seedling stage)	GS 21 (Tillering stage)	GS 32 (stem elongation stage)
200A12	Fungal biomass	−22.9	−65.6	−82.9
	Uredinia number	−30.7	−66.9	−93.6
	Rust LP	+7.9	+2.3	+21.3
182Q20	Fungal biomass	−70.0	−77.2	−86.0
	Uredinia number	−60.7	−77.1	−87.9
	Rust LP	+29.8	+38.4	+44.4

### Uredinia development at booting stage

**FIGURE 3:**
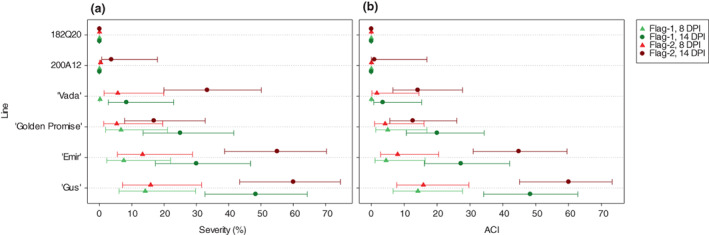
(a) Dot‐plot of mean severity (%) and (b) average coefficient of infection (ACI), for the six barley test lines, on Flag–1 and −2 leaves, at 8 and 14 days post inoculation with *Puccinia hordei* at adult plant stage. Error bars are 95% confidence limits for the means. Lines are in increasing order of overall mean severity/CI. Note that the upper limit for a mean of ‘0’ is difficult to obtain, so is not shown.

For both disease severity and CI, there were on average strong differences amongst the barley lines (*p* < 0.001 for severity and CI, for the barley line main effect). Differences between Flag‐1 and ‐2 leaves were weaker (*p* = 0.011 for severity and *p* = 0.072 for CI, for the leaf main effect), with severity and CI tending to be higher on Flag‐2 leaf (Figure 3). However, for severity, the leaf effect varied somewhat between barley lines (*p* = 0.072 for the leaf by barley line interaction). For the most susceptible cultivars ‘Emir’ and ‘Gus’, both Flag‐1 and ‐2 leaves had about 15% uredinia coverage at 8 DPI, which increased to 50%–90% at 14 DPI (Figure 3). In addition, ‘Gus’ had the highest ACI scores at 14 DPI for both Flag‐1 and ‐2 leaves followed by ‘Emir’ (Figure 3). Cultivars ‘Golden Promise’ and ‘Vada’ had lower uredinia coverage and less susceptible responses with ACI scores ranging from 0.1 to 5.0 at 8 DPI and 3.5 to 20.0 at 14 DPI (Figure 3). A notable result was that the ACI scores of ‘Emir’ and ‘Vada’ at 8 DPI generally accounted *f* or 15% of their scores at 14 DPI, while cultivars ‘Gus’ and ‘Golden Promise’ reached 25%–33% of their final scores at 8 DPI. This indicted that ‘Emir’ had a prolonged rust LP at adult plant stage similar to the partially resistant cultivar ‘Vada’. As for the two ILs, most of the leaves assessed had no visible symptoms (no uredinia), except 200A12 had very low disease severities (2%–10%) on a few inoculated leaves. According to the ACI scores for high, moderate, low and lack of APR, ‘Gus’ and ‘Emir’ were considered to be susceptible at the adult plant stage. Cultivars ‘Golden Promise’ and ‘Vada’ showed low–moderate and moderate–high APR, respectively, to *P. hordei*. The two ILs 200A12 and 182Q20 were classified into a highly resistant group at adult plant stage.

## DISCUSSION

In the current study, GS played the most important role in the expression of resistance genes *Rph22* and *Rph26* to *P. hordei*. The two ILs 182Q20 and 200A12 showed susceptible ITs at seedling stage (GS 12) to the rust pathogen, but increased in resistance (i.e. reduced visual scores and fungal biomass, and increased LPs) as the plants developed. High level of resistance was observed in both ILs at adult plant stage. A study, which evaluated an integrated seedling and adult plant phenotyping method against leaf rust, had shown that responses from the adult plants in the integrated method only had minor/insignificant differences compared with those phenotypic responses from independent adult plant experiment, and at levels similar to those observed in the field (Riaz et al., 2016). The effect of taking the seedlings through to adult plants on inducing resistance was expected to be minimal. Thus, the two genes derived from *H. bulbosum* were confirmed to have typical APR phenotypes in *H. vulgare* backgrounds against *P. hordei*.

Although 182Q20 had a susceptible IT (3+) at GS 12, the resistance conferred by *Rph22* resulted in a more than 60% reduction in uredinia number and fungal biomass when compared with its barley background ‘Golden Promise’. The resistance expression became stronger with further reductions in uredinia number and fungal biomass and ITs as plants developed. Similarly, 200A12 showed a more subtle resistance at seedling stage, but the resistance became stronger as plants developed further. The contrasting phenotypes to *P. hordei* at seedling and post‐seedling stages for the two ILs suggest that relying on phenotypic symptoms (i.e. IT) at seedling stage alone can prevent identification of quantitative or partial resistance at post‐seedling stages.

The increase resistance over plant growth (reflected by the prolonged LPs of uredinia formation) in both ILs relative to their barley parents (Table 2) has confirmed the influence of plant development stages on rust latency as previously reported (Parlevliet, 1975). In this study, IL 200A12 was expected to reach greater than 7.9% longer LP relative to ‘Emir’ but only resulted in 2.3% increase. This could be caused by the influence of varying urediniospore densities deposited on the leaf surfaces during inoculation. The mean urediniospore density (50.0 spores cm^−2^ with 88.3% germination rate) captured on the water agar slides during inoculation at GS 21 was higher than those captured at GS 12 (18.1 spores cm^−2^ with 85.7% germination rate). Decreased rust LP with higher spore density has been reported previously (Roelfs et al., 1992; Teng & Close, 1978). Thus, the higher disease pressure at GS 21 probably resulted in shorter LP of the rust pathogen. Prolonged LPs of *P. hordei* in ILs 200A12 and 182Q20 were first reported by Pickering et al. (2004) in which ILs 200A12 and 182Q20 (coded as ‘E‐1HL’ and ‘G‐2HL‐b’, respectively) were identified to have 16.2% and 43.5% longer rust LPs than the respective ‘Emir’ (6.29 days) and ‘Golden Promise’ (6.40 days) at seedling stage. In the current study, similar results were obtained at stem elongation stage (GS 32). Apart from disease pressure, environmental factors including moisture for initial disease development, post‐inoculation temperature and light intensity may directly or indirectly influence fungal LP in cereals (Denissen, 1991; Pfender, 2001; Rapilly, 1979; Shearer & Zadoks, 1972; Teng & Close, 1978; Tomerlin et al., 1983). Experimental differences in these factors might have resulted in the shorter LPs of uredinia formation in the two ILs at seedling stage in the current study.

As a quantitative measurement similar to uredinia count, fungal biomass assessment has proven to be a useful tool for quantifying phenotypic expression conferred by the two resistance genes *Rph22* and *Rph26*. The net internal fungal growth related strongly to the final uredinia counts at GS 21 (*r* = 0.95) and GS 32 (*r* = 0.99) but not at GS 12 (*r* = 0.78). At GS 12, the most susceptible barley cultivars ‘Emir’ and ‘Gus’ swapped in their rankings between the two assessments. The IL 200A12 with the third highest mean uredinia number (54.2) only had the second lowest fluorescence reading (467.2 FU) after the most resistant line 182Q20 (166.9 FU). Meanwhile, cultivars ‘Golden Promise’ and ‘Vada’ with lower final uredinia numbers were all observed to have more severe internal fungal growth than the IL 200A12. In other words, apart from the most resistant line 182Q20, the IL 200A12 and ‘Emir’ were identified to be more ‘resistant’ to *P. hordei* at seedling stage in terms of internal fungal biomass assessment compared with the external assessments. The internal resistance expressed by ‘Emir’, 200A12 and 182Q20 to *P. hordei* at seedling stage was also reported by Pickering et al. (2004) based on another assessment of early absorbed infection units. The authors evaluated the infection units on infected leaves at 4 DPI using UV microscopy technique and found high percentages of early absorbed infection units in ‘Emir’ (10%), 200A12 (26%) and 182Q20 (51%) by taking a partially resistant control ‘Vada’ (12%) as reference. The infection units that had formed primary infection hyphae but failed to have six mycelial branches were classified as ‘early absorbed’, and high percentages of early absorbed infection units, which indicated strong suppression of the advancement of rust pathogen inside leaves, were associated with high levels of partial resistance (Pickering et al., 2004). In addition, most early absorbed infection units in ‘Emir’ (93%), 200A12 (72%) and 182Q20 (86%) were not associated with yellow autofluorescent plant cells (i.e. necrosis), indicating that the resistance conferred by these lines at seedling stage were probably not based on hypersensitivity (Pickering et al., 2004). In comparison to uredinia count, the fungal biomass analysis applied in the current study was considered a comprehensive measurement to evaluate various aspects of barley resistance responses, such as uredinia number, size, sporulation and internal rust development to *P. hordei*. All these aspects were assessed together by means of quantifying the chitin content of the rust pathogen in infected plant tissues, and the disease development was then measured in fluorescent units at an integral level.

In the current study, disease symptoms expressed on ‘Gus’ were used as a susceptible reference to standardize leaf rust scores. This would remove any confounding variation caused by inoculation or environmental conditions. Although having less final uredinia at seedling stage, ‘Gus’ appeared to have more severe rust infection (i.e. rust fungal biomass) than ‘Emir’ due to the larger uredinial size and greater sporulation on its leaves. The subtle disease resistance on ‘Emir’ observed from the collective fungal biomass analysis, early absorbed infection units at seedling stage and prolonged rust LP at adult plant stage, could be attributed to the expression of APR gene *Rph20* (Hickey et al., 2012). Some studies have shown that ‘Emir’ have highly effective APR against *P. hordei* (Dracatos et al., 2015; Golegaonkar et al., 2009). *Rph20* has been reported to be temperature sensitive and most effective at cooler temperatures (18 ± 2°C) (Singh et al., 2013). The temperatures (22 ± 2°C) used in the current study might not be optimal for its expression. The phenotype expression of ‘Emir’ with the same rust isolate under lower temperatures will be investigated in future studies. Furthermore, it was possible that the different seed sources of ‘Emir’ used in the current and other studies (Dracatos et al., 2015; Golegaonkar et al., 2009) might have contributed to the contradictory responses. Moreover, ITs and APR ratings may vary when comparing trials performed under controlled environment (current study) to field conditions as in the study by Golegaonkar et al. (2009). Future studies will also include assessing the phenotype responses of the test lines under field conditions.

Although non‐host resistance is considered more durable than host resistance, it has been hypothesized that the components contributing to non‐host resistance are no different from those used in host immune systems against adapted pathogens (Dinh et al., 2020; Panstruga & Moscou, 2020). In the current study, the two genes *Rph22* and *Rph26* were characterized to have typical APR phenotypes against *P. hordei*. This suggests that the two components separated from the non‐host resistance machinery of *H. bulbosum* might have the same defence mechanisms against barley leaf rust as APR genes derived from barley plants. *Rph22* was successfully cloned and shown to encode a receptor‐like kinase (RLK) protein (Wang et al., 2019). The multigene family of RLKs has been widely reported in many host plant species to confer resistance against adapted pathogens, indicating that RLK protein is not specific to non‐host resistance (Cole & Diener, 2013; Duriez et al., 2019; Jinrong et al., 2008; Saintenac et al., 2018; Song et al., 1995). Although the major group of nucleotide‐binding, leucine‐rich repeat (NB‐LRR) protein genes has not been isolated in leaf rust resistant *H. bulbosum* ILs, the typical hypersensitive resistance normally mediated by NB‐LRR genes were identified in many *H. bulbosum* ILs against barley leaf rust (Pickering et al., 2004). A recent study transferred a gene cassette of five stem rust resistance genes, *Sr22*, *Sr35*, *Sr45*, *Sr50* and *Sr55*, into bread wheat (*Triticum aestivum*) to confer immune‐like resistance, at both seedling (IT, 0) and adult plant (average disease severity, 0%) stages, against highly virulent *Puccinia graminis* isolates from around the world (Luo et al., 2021). Due to the extensive deployment of these resistances as single genes, a *P. graminis* isolate virulent to *Sr35*, *Sr45* and *Sr50* has already been detected even before stacking these genes together (Bhattacharya, 2017). Despite this, pyramiding multiple resistance genes, including overcome ones, can still reduce the chances of a new pathotype with corresponding mutations from developing, making the host resistance more effective and more durable. Future stacks of undeployed resistance genes were considered to be extremely durable against *P. graminis* (Schafer & Roelfs, 1985). Based on the findings above, it may be easy to understand the durable and uniform resistance of *H. bulbosum*, that is, non‐host resistance, against *P. hordei* isolates if *H. bulbosum* is considered as a stack of multiple resistance genes that have never been challenged singly with *P. hordei*.

Barley germplasm with durable resistance to *P. hordei* is an important objective for breeding programmes. However, due to the presence of new pathotypes, most of the identified qualitative resistance genes have already been overcome by *P. hordei* (Park et al., 2015). Although quantitative or APR is considered more durable, it has been difficult to study and utilize because of their polygenic nature and quantitative phenotypes (St.Clair, 2010). In the present study, the two barley APR genes *Rph22* and *Rph26* to *P. hordei* were well characterized, and the results provided an increased understanding on their effectiveness against rust infection. This information is vital for future deployment of these two resistance genes in barley breeding programmes as one of the tools for combating leaf rust. In addition, the collective knowledge of plant developmental stage on the expression of APR genes and the effectiveness of using multiple assessment scales underpin future identification and characterization of quantitative resistance or APR genes against other rust diseases.

## CONFLICT OF INTEREST

On behalf of all authors, the corresponding author states that there is no conflict of interest.

## References

[jam15710-bib-0001] Anikster, Y. (1989) Host specificity versus plurivority in barley leaf rusts and their microcyclic relatives. Mycological Research, 93, 175–181.

[jam15710-bib-0002] Ayliffe, M. , Periyannan, S.K. , Feechan, A. , Dry, I. , Schumann, U. , Lagudah, E. et al. (2014) Simple quantification of in planta fungal biomass. In: Birch, P. , Jones, J.T. & Bos, J.I.B. (Eds.) Plant‐pathogen interactions: methods and protocols. Totowa, NJ: Humana Press, pp. 159–172. 10.1007/978-1-62703-986-4_13 24643560

[jam15710-bib-0003] Bettgenhaeuser, J. , Gilbert, B. , Ayliffe, M. & Moscou, M.J. (2014) Nonhost resistance to rust pathogens – a continuation of continua. Frontiers in Plant Science, 5, 664. 25566270 10.3389/fpls.2014.00664 PMC4263244

[jam15710-bib-0004] Bhattacharya, S. (2017) Deadly new wheat disease threatens Europe's crops. Nature (Lond), 542, 145–146. 28179687 10.1038/nature.2017.21424

[jam15710-bib-0005] Clifford, B.C. (1985) Barley Leaf Rust. Barley leaf rust. Cereal rusts, 2, 173–205.

[jam15710-bib-0006] Cole, S.J. & Diener, A.C. (2013) Diversity in receptor‐like kinase genes is a major determinant of quantitative resistance to *Fusarium oxysporum* f.sp. matthioli. The New Phytologist, 200, 172–184. 23790083 10.1111/nph.12368

[jam15710-bib-0007] Denissen, C.J.M. (1991) Influence of race and post infection temperature on two components of partial resistance to wheat leaf rust in seedlings of wheat. Euphytica, 58, 13–20.

[jam15710-bib-0008] Derevnina, L. , Singh, D. & Park, R.F. (2013) Identification and characterization of seedling and adult plant resistance to *Puccinia hordei* in Chinese barley germplasm. Plant Breeding, 132, 571–579.

[jam15710-bib-0009] Dinh, H.X. , Singh, D. , Periyannan, S. , Park, R.F. & Pourkheirandish, M. (2020) Molecular genetics of leaf rust resistance in wheat and barley. Theoretical and Applied Genetics, 133, 2035–2050. 32128617 10.1007/s00122-020-03570-8

[jam15710-bib-0010] Dracatos, P.M. , Singh, D. , Bansal, U. & Park, R.F. (2015) Identification of new sources of adult plant resistance to *Puccinia hordei* in international barley (*Hordeum vulgare* L.) germplasm. European Journal of Plant Pathology, 141, 463–476.

[jam15710-bib-0011] Duriez, P. , Vautrin, S. , Auriac, M.‐C. , Bazerque, J. , Boniface, M.‐C. , Callot, C. et al. (2019) A receptor‐like kinase enhances sunflower resistance to *Orobanche cumana* . Nature Plants, 5, 1211–1215. 31819219 10.1038/s41477-019-0556-z

[jam15710-bib-0012] Fetch, T., Jr. , Johnston, P.A. & Pickering, R. (2009) Chromosomal location and inheritance of stem rust resistance transferred from *Hordeum bulbosum* into cultivated barley (*H. vulgare*). Phytopathology, 99, 339–343. 19271974 10.1094/PHYTO-99-4-0339

[jam15710-bib-0013] Golegaonkar, P.G. , Singh, D. & Park, R.F. (2009) Evaluation of seedling and adult plant resistance to *Puccinia hordei* in barley. Euphytica, 166, 183–197.

[jam15710-bib-0014] Griffey, C.A. , Das, M.K. , Baldwin, R.E. & Waldenmaier, C.M. (1994) Yield losses in winter barley resulting from a new race of *Puccinia hordei* in North America. Plant Disease, 78, 256–260.

[jam15710-bib-0015] Hickey, L.T. , Lawson, W. , Platz, G.J. , Dieters, M. & Franckowiak, J. (2012) Origin of leaf rust adult plant resistance gene Rph20 in barley. Genome, 55, 396–399. 22533489 10.1139/g2012-022

[jam15710-bib-0016] Jinrong, W. , Xue‐Cheng, Z. , David, N. , Katrina, M.R. , Steve, C. , Sung‐yong, K. et al. (2008) A LysM receptor‐like kinase plays a critical role in chitin signaling and fungal resistance in Arabidopsis. The Plant Cell, 20, 471–481. 18263776 10.1105/tpc.107.056754 PMC2276435

[jam15710-bib-0017] Johnston, P.A. , Niks, R.E. , Meiyalaghan, V. , Blanchet, E. & Pickering, R. (2013) Rph22: mapping of a novel leaf rust resistance gene introgressed from the non‐host *Hordeum bulbosum* L. into cultivated barley (*Hordeum vulgare* L.). Theoretical and Applied Genetics, 126, 1613–1625. 23467993 10.1007/s00122-013-2078-9

[jam15710-bib-0018] Jones, J.D.G. & Dangl, J.L. (2006) The plant immune system. Nature, 444, 323–329. 17108957 10.1038/nature05286

[jam15710-bib-0019] Kumar, S. , Phogat, B. , Vikas, V. , Sharma, A. , Saharan, M. , Singh, A.K. et al. (2019) Mining of Indian wheat germplasm collection for adult plant resistance to leaf rust. PLoS One, 14, e0213468. 30921352 10.1371/journal.pone.0213468 PMC6438482

[jam15710-bib-0020] Kushalappa, A.C. , Yogendra, K.N. & Karre, S. (2016) Plant innate immune response: Qualitative and quantitative resistance. Critical Reviews in Plant Sciences, 35, 38–55.

[jam15710-bib-0021] Lee, Y. , Nelder, J.A. & Pawitan, Y. (2006) Generalized linear models with random effects: unified analysis via H‐likelihood. London: Chapman & Hall/CRC Press.

[jam15710-bib-0022] Lo Iacono, G. , van den Bosch, F. & Paveley, N. (2012) The evolution of plant pathogens in response to host resistance: Factors affecting the gain from deployment of qualitative and quantitative resistance. Journal of Theoretical Biology, 304, 152–163. 22483999 10.1016/j.jtbi.2012.03.033

[jam15710-bib-0023] Luo, M. , Xie, L. , Chakraborty, S. , Wang, A. , Matny, O. , Jugovich, M. et al. (2021) A five‐transgene cassette confers broad‐spectrum resistance to a fungal rust pathogen in wheat. Nature Biotechnology, 39, 561–566. 10.1038/s41587-020-00770-x 33398152

[jam15710-bib-0024] McCullagh, P. & Nelder, J.A. (1989) Generalized linear models. London: Chapman & Hall.

[jam15710-bib-0025] McIntosh, R.A. , Wellings, C.R. & Park, R.F. (1995) Wheat rusts: an atlas of resistance genes. Melbourne: CSIRO Publishing.

[jam15710-bib-0026] Murray, G.M. & Brennan, J.P. (2010) Estimating disease losses to the Australian barley industry. Australasian Plant Pathology, 39, 85–96.

[jam15710-bib-0027] Panstruga, R. & Moscou, M.J. (2020) What is the molecular basis of nonhost resistance? Molecular Plant‐Microbe Interactions, 33, 1253–1264. 32808862 10.1094/MPMI-06-20-0161-CR

[jam15710-bib-0028] Park, R.F. (2003) Pathogenic specialization and pathotype distribution of *Puccinia hordei* in Australia, 1992 to 2001. Plant Disease, 87, 1311–1316. 30812545 10.1094/PDIS.2003.87.11.1311

[jam15710-bib-0029] Park, R.F. , Golegaonkar, P.G. , Derevnina, L. , Sandhu, K.S. , Karaoglu, H. , Elmansour, H.M. et al. (2015) Leaf rust of cultivated barley: Pathology and control. Annual Review of Phytopathology, 53, 565–589. 10.1146/annurev-phyto-080614-120324 26047566

[jam15710-bib-0030] Parlevliet, J.E. (1975) Partial resistance of barley to leafrust, *Puccinia hordei*. I. Effect of cultivar and development stage on latent period. Euphytica, 24, 21–27.

[jam15710-bib-0031] Payne, R. , Welham, S. & Harding, S. (2017) A guide to REML in Genstat. Hemel Hempstead, Hertfordshire, UK: VSN International.

[jam15710-bib-0032] Peterson, R.F. , Campbell, A.B. & Hannah, A.E. (1948) A diagrammatic scale for estimating rust intensity on leaves and stems of cereals. Canadian Journal of Research, 26, 496–500.

[jam15710-bib-0033] Pfender, W.F. (2001) A temperature‐based model for latent‐period duration in stem rust of perennial ryegrass and tall fescue. Phytopathology, 91, 111–116. 18944285 10.1094/PHYTO.2001.91.1.111

[jam15710-bib-0034] Pickering, R. , Niks, R.E. , Johnston, P.A. & Butler, R.C. (2004) Importance of the secondary genepool in barley genetics and breeding, 2: Disease resistance, agronomic performance and quality. Czech Journal of Genetics and Plant Breeding, 40, 79–85.

[jam15710-bib-0035] Pickering, R.A. , Hill, A.M. , Michel, M. & Timmerman‐Vaughan, G.M. (1995) The transfer of a powdery mildew resistance gene from *Hordeum bulbosum* L to barley (*H. vulgare* L.) chromosome 2 (2I). Theoretical and Applied Genetics, 91, 1288–1292. 24170060 10.1007/BF00220943

[jam15710-bib-0036] Pickering, R.A. , Malyshev, S. , Künzel, G. , Johnston, P.A. , Korzun, V. , Menke, M. et al. (2000) Locating introgressions of *Hordeum bulbosum* chromatin within the *H. vulgare* genome. Theoretical and Applied Genetics, 100, 27–31.

[jam15710-bib-0037] Pickering, R.A. , Steeffenson, B.J. , Hill, A.M. & Borovkova, I. (1998) Association of leaf rust and powdery mildew resistance in a recombinant derived from a *Hordeum vulgare × Hordeum bulbosum* hybrid. Plant Breeding, 117, 83–84.

[jam15710-bib-0038] Rapilly, F.J. (1979) Yellow rust epidemiology. Annual Review of Phytopathology, 17, 59–73.

[jam15710-bib-0039] Riaz, A. , Periyannan, S. , Aitken, E. & Hickey, L. (2016) A rapid phenotyping method for adult plant resistance to leaf rust in wheat. Plant Methods, 12, 1–10. 26941830 10.1186/s13007-016-0117-7 PMC4776422

[jam15710-bib-0040] Roelfs, A.P. , Singh, R.P. & Saari, E.E. (1992) Rust diseases of wheat: concepts and methods of disease management. Mexico: Centro Internacional de Mejoramiento de Maiz y Trigo, vi + 81 pp.

[jam15710-bib-0041] Ross, G.J.S. (1984) Parallel model analysis: fitting non‐linear models to several sets of data. In: Havránek, T. , Šidák, Z. & Novák, M. (Eds.) Compstat 1984. Heidelberg: Physica‐Verlag, pp. 458–463.

[jam15710-bib-0042] Saintenac, C. , Lee, W.‐S. , Cambon, F. , Rudd, J.J. , King, R.C. , Marande, W. et al. (2018) Wheat receptor‐kinase‐like protein Stb6 controls gene‐for‐gene resistance to fungal pathogen *Zymoseptoria tritici* . Nature Genetics, 50, 368–374. 29434355 10.1038/s41588-018-0051-x

[jam15710-bib-0043] Sarris, P.F. , Duxbury, Z. , Huh, S.U. , Ma, Y. , Segonzac, C. , Sklenar, J. et al. (2015) A plant immune receptor detects pathogen effectors that target WRKY transcription factors. Cell, 161, 1089–1100. 26000484 10.1016/j.cell.2015.04.024

[jam15710-bib-0044] Schafer, J.F. & Roelfs, A.P. (1985) Estimated relation between numbers of urediniospores of *Puccinia graminis* f. sp. tritici and rates of occurrence of virulence. Phytopathology, 75, 749–750.

[jam15710-bib-0045] Shearer, B.L. & Zadoks, J.C. (1972) The latent period of *Septoria nodorum* in wheat. 1. The effect of temperature and moisture treatments under controlled conditions. Netherlands Journal of Plant Pathology, 78, 231–241.

[jam15710-bib-0046] Singh, D. , Macaigne, N. & Park, R.F. (2013) Rph20: adult plant resistance gene to barley leaf rust can be detected at early growth stages. European Journal of Plant Pathology, 137, 719–725.

[jam15710-bib-0047] Song, W.‐Y. , Wang, G.‐L. , Chen, L.‐L. , Kim, H.‐S. , Pi, L.‐Y. , Holsten, T. et al. (1995) A receptor kinase‐like protein encoded by the rice disease resistance gene, Xa21. Science (Wash D C), 270, 1804–1806. 10.1126/science.270.5243.1804 8525370

[jam15710-bib-0048] St.Clair, D.A. (2010) Quantitative disease resistance and quantitative resistance loci in breeding. Annual Review of Phytopathology, 48, 247–268. 10.1146/annurev-phyto-080508-081904 19400646

[jam15710-bib-0049] Szigat, G. & Pohler, W. (1982) *Hordoum bulbosum* × *H. vulgare* hybrids and their backcrosses with cultivated barley. Cereal Research Communications, 10, 73–78.

[jam15710-bib-0050] Teng, P.S. & Close, R.C. (1978) Effect of temperature and uredinium density on urediniospore production, latent period, and infectious period of *Puccinia hordei* Otth. New Zealand Journal of Agricultural Research, 21, 287–296.

[jam15710-bib-0051] Tomerlin, J.R. , Eversmeyer, M.G. , Browder, L.E. & Kramer, C.L. (1983) Temperature and host effects on latent and infectious periods and on urediniospore production of *Puccinia recondita* f. sp. tritici. Journal of Phytopathology, 73, 414–419.

[jam15710-bib-0052] VSN International Ltd . (2015) Genstat reference manual (release 18), part 3: procedures. Hemel Hempstead, Hertfordshire, UK: VSN International.

[jam15710-bib-0053] Wang, Y. , Subedi, S. , de Vries, H. , Doornenbal, P. , Vels, A. , Hensel, G. et al. (2019) Orthologous receptor kinases quantitatively affect the host status of barley to leaf rust fungi. Nature Plants, 5, 1129–1135. 31712760 10.1038/s41477-019-0545-2

[jam15710-bib-0054] Wendler, N. , Mascher, M. , Himmelbach, A. , Johnston, P. , Pickering, R. & Stein, N. (2015) Bulbosum to go: a toolbox to utilize *Hordeum vulgare*/*bulbosum* introgressions for breeding and beyond. Molecular Plant, 8, 1507–1519. 25983208 10.1016/j.molp.2015.05.004

[jam15710-bib-0055] Wright, A.C. & Gaunt, R.E. (1992) Disease‐yield relationship in barley. Plant Pathology, 41, 676–687.

[jam15710-bib-0056] Xu, J. & Kasha, K.J. (1992) Transfer of a dominant gene for powdery mildew resistance and DNA from *Hordeum bulbosum* into cultivated barley (*H. vulgare*). Theoretical and Applied Genetics, 84, 771–777. 24201473 10.1007/BF00227383

[jam15710-bib-0057] Yu, X. , Kong, H.Y. , Meiyalaghan, V. , Casonato, S. , Chng, S. , Jones, E.E. et al. (2018) Genetic mapping of a barley leaf rust resistance gene Rph26 introgressed from *Hordeum bulbosum* . Theoretical and Applied Genetics, 131, 2567–2580. 30178277 10.1007/s00122-018-3173-8

[jam15710-bib-0058] Zadoks, J.C. , Chang, T.T. & Konzak, C.F. (1974) A decimal code for the growth stages of cereals. Weed Research, 14, 415–421.

